# A new instrument to predict smoking cessation among patients with chronic obstructive pulmonary disease: an observational longitudinal study of the Trying To Quit smoking questionnaire

**DOI:** 10.1038/npjpcrm.2016.13

**Published:** 2016-04-14

**Authors:** Lena Lundh, Hassan Alinaghizadeh, Lena Törnkvist, Hans Gilljam, Maria Rosaria Galanti

**Affiliations:** 1 Academic Primary Health Care Centre, Department of Neurobiology, Care Sciences and Society (NVS), Karolinska Institutet, Huddinge, Sweden; 2 Department of Public Health Science, Karolinska Institutet, Huddinge, Sweden

## Abstract

The Trying To Quit smoking questionnaire (TTQ), was developed to measure pressure-filled mental states, use of destructive pressure-relief strategies and ambivalent thoughts about quitting smoking among patients with COPD. The aim of this study was to evaluate whether the TTQ (available in an extended and in a reduced version) can be used to predict smoking cessation outcomes in smokers with COPD. As higher TTQ scores indicate higher degree of psychological distress, we hypothesised that TTQ scores at baseline would be negatively correlated with the probability of making a quit attempt, reducing the intensity of smoking and achieving complete abstinence during the 3 months. Smokers with COPD were recruited during planned or unplanned visits to primary healthcare centres, and 109 completed the TTQ at baseline and 85% participated in the follow-up after 3 months. Logistic regression was used to measure the association between the original (19 items) and the brief (14 item) version of TTQ scores and three outcomes: making at least one quit attempt, reducing the intensity of smoking and achieving complete abstinence. In a primary analysis among all the participants higher total score in the original version of TTQ was significantly associated with a lower probability of quit attempts. In a secondary analysis of subgroups of patients classified according to their readiness to quit, high TTQ scores at baseline were associated with lower probability of complete abstinence among patients not ready to quit (adjusted odds ratio (OR)=0.72; 95% confidence interval (CI)=0.53–0.99). Among patients ready to quit, high score on pressure-filled mental states was associated with lower probability of quit attempts (OR=0.78; 95% CI=0.66–0.94) but with higher probability of reduced smoking (OR=1.32; 95% CI=1.05–1.66). Ambivalent thoughts were associated with lower probability of all outcomes, but estimates were not statistically significant. Destructive coping strategies were inconsistently associated with the outcomes. TTQ in its original version and two of its subscales predicted smoking cessation outcomes in the anticipated direction. Therefore, this instrument may be useful in tailoring smoking cessation counselling for patients with COPD.

## Introduction

Cigarette smoking is the primary cause of chronic obstructive pulmonary disease (COPD) and its progression. Nevertheless, smoking prevalence is high in COPD (38–77%) patients.^1^ Every episode of smoking abstinence can decrease the risk of new exacerbations, the most important determinant of disease progression.^2^ It is therefore important for smokers with COPD to continue to try to quit and to improve the success of each quit attempt.

Most smoking cessation programs for patients with COPD do not differ from those developed for other smokers^3^ despite indications that a diagnosis of COPD prompts massive cessation support.^4,5^ The large-scale Lung Health Study, in which such intensive support was provided, showed that about one-third of the COPD patients could reach sustained abstinence. The Lung Health Study also proved that improvements in morbidity and mortality could be reached.^6^

In 2007, an expert panel published guidelines for smoking cessation in patients with respiratory illness.^7^ To provide the recommended smoking cessation help, it is important to adopt individualised treatment strategies. For instance, healthcare professionals should be able to identify patients who are less likely to make a quit attempt and/or less likely to succeed and to explore the challenges faced by such patients. Even among smokers committed to quitting, relapses may be triggered both by nicotine dependence and by non-nicotine cues,^8^ such as seeing a pack of cigarettes or finding oneself in situations associated with smoking. It is therefore necessary to consider the specific triggers that lead people with COPD to relapse.^8^ The 2007 expert panel concluded that there was a lack of research about individualised treatment strategies for people with respiratory illness.^7^

The Trying To Quit smoking questionnaire (TTQ)^9^ was developed to facilitate individualised cessation support for smokers with COPD.^10^ The questionnaire assesses difficulties and mental processes that people with COPD may experience, and coping strategies that they may use during the process of smoking cessation.^10^ The factors of importance in the success or failure of a quit attempt included pressure-filled mental states, ambivalent thoughts and destructive pressure-relief strategies. The questionnaire was found internally consistent and reliable in previous psychometric analyses.^9^

The aim of this study was to evaluate whether the TTQ (available in an extended and in a reduced version) can be used to predict smoking cessation outcomes in smokers with COPD. As higher TTQ scores indicate higher degrees of psychological distress, we hypothesised that TTQ scores at baseline would be negatively correlated with the probability of three outcomes related to smoking cessation: making a quit attempt, reducing the intensity of smoking and achieving complete abstinence for 3 months.

## Results

### Characteristics of the study population

Baseline characteristics of the study population are presented in Tables 1 and 2. The majority of the participants were women who were on average older than men (66 vs. 62 years; *P*<0.02), but there were no other statistically significant differences between the sexes. The highest educational level achieved by almost half of the participants was elementary school, and more than 50% were married or cohabited with a partner.

The average severity of COPD, classified using the categories of the Global Initiative for chronic obstructive lung disease,^11^ was moderate. Nine (10%) participants were diagnosed with mild COPD, 62 participants (64% of the total population) with moderate COPD, 20 (22%) with severe COPD and six (6%) with very severe COPD. There was no correlation between cigarette dependence scores and disease severity (Spearman correlation coefficient −0.16, *P*=0.11).

The participants had been smoking for an average of 45 years. Most of the participants were in the process of quitting, but 38% reported no intention to quit in the near future. There were no statistically significant differences at baseline between the participants who were not ready to try to quit and those who were ready to try to quit (Tables 1 and 3).

A total of 38% of patients received, at baseline, brief advice on quitting (less than 5 min, including written information about quitting), and an equal percentage received smoking cessation counselling (recommendation tailored to person’s age, health and risk level and could include pharmacotherapy). Eighteen percent were referred to the Swedish Tobacco Quit line. Four percent of patients were offered advanced smoking cessation counselling (theory-based counselling in combination with pharmacological treatment and follow-ups). One-third of all the participants had more than three counselling meetings. The patients who were ready to try to quit smoking and who were offered counselling had a higher total TTQ score (mean 27.90, s.d.=5.21) than those offered brief advice or referred to the quit line (mean 26.62, s.d.=4.59), but the difference was not statistically significant (*P* value=0.29). Among patients not ready to try to quit smoking, there were no differences in the total TTQ scores between those offered counselling (mean 28.53, s.d.=5.59) and those offered brief advice or referred to the quit line (mean 28.71, s.d.=4.16). Ninety-four of the 109 participants (85%) took part in the follow-up 3 months after baseline. At follow–up, 17 participants had quit smoking completely, 36 participants had made at least one quit attempt lasting 24 h and 31 participants had decreased the number of cigarettes smoked per day by at least 50% (Table 4).

### Analysis of total scores on the TTQ

The association between 19-item and 14-item TTQ scores and each of the smoking cessation outcomes is shown in Table 4. The results are also presented separately for patients who were ready to try to quit and those who were not ready to try to quit. In the primary analyses of all the participants, the higher total 19-item TTQ score was significantly positively associated with a lower probability of quit attempts, whereas it was not significantly associated with a 50% reduction in the amount of smoking between baseline and follow-up or with achieving complete abstinence during the 7 days before follow-up. The results did not change after adjustment for age, education, family situation, time to diagnosis, severity of disease, smoking habits, risky alcohol use, smoking cessation counselling, and anxiety and depression. The secondary analysis of subgroups of patients according to their readiness to quit revealed that the negative associations between the TTQ scores and the cessation outcomes were present only among patients who were not ready to try to quit. The total TTQ score based on the 14 items was not significantly associated with the probability of quit attempts, 50% reduction in the intensity of smoking or achieving complete abstinence.

### Analysis of subscale scores on the 14-item TTQ

Among the participants, there was no association between scores in any of the three TTQ subscales and smoking cessations outcomes. A secondary analysis of participants categorised by readiness to quit at baseline revealed that the proportion making a quit attempt, reducing the intensity of smoking and achieving complete abstinence did not differ between those ready and not ready to try to quit (Table 4). In a separate analysis of the three TTQ subscales, development of pressure-filled mental states was significantly associated with a lower probability of making a quit attempt among the participants ready to try to quit smoking, but the use of destructive pressure-relief strategies was not. In the group of participants not ready to try to quit, higher scores indicative of pressure-filled mental states increased the probability of reducing the intensity of smoking (Table 4). Higher scores in the subscale indicating destructive pressure-relief strategies also predicted an increased probability of smoking cessation outcomes (Table 4, right columns).

Having ambivalent thoughts was not significantly associated with any of the three outcomes in either group. However, among patients not ready to try to quit, increasing scores in this subscale were associated (not significantly) with 34% decreased probability of achieving complete abstinence at follow-up.

The total TTQ score did not predict the transition to readiness to quit smoking among patients not ready to try to quit at baseline. Ambivalent thoughts were negatively associated with this transition, albeit not significantly (odds ratio (OR)=0.78, 95% confidence interval (CI)=-0.54–1.13).

## Discussion

### Main findings

In this study, the TTQ, designed to assess obstacles to successful smoking cessation in patients with COPD, predicted smoking cessation outcomes in the anticipated direction after controlling for potential confounding factors, including the treatment received at enrolment. In the primary analysis, the association between the original 19-item TTQ and cessation outcomes was consistent for all chosen end points, from attempting to quit to achieving complete short-term abstinence, although the estimates did not always attain conventional statistical significance.

The predictive ability of the brief 14-item TTQ was lower, and its subscales were associated with the three different outcomes in a way that varied by patients’ readiness to quit.

In the secondary analysis of the three subscales in the 14-item questionnaire, only development of pressure-filled mental states predicted making at least a quit attempt among patients ready to try to quit at baseline. Contrary to expectations, the development of pressure-filled mental states was associated with a higher probability of reducing the intensity of smoking in the group not ready to try to quit.

The second subscale (use of destructive pressure-relief strategies) was inconsistently associated with the smoking cessation outcomes, and, also contrary to expectations, even seemed to predict a higher probability of achieving complete abstinence at follow-up in both the subgroups of patients—those ready to try to quit and those not ready to try to quit.

The third subscale (‘ambivalent thoughts’) was not significantly associated with cessation outcomes in either group. However, among patients not ready to try to quit at baseline, there was some indication that increasing scores in this subscale may be associated with achieving complete abstinence.

It is not easy to reconcile these apparently contradictory findings. One explanation may lie in the unexpected finding that the short-term rate of smoking cessation outcomes was practically identical in the two groups, despite baseline differences in self-reported readiness to make a quit attempt.

### Interpretation of findings in relation to previously published work

The development of pressure-filled mental states included reports of self-critical statements and constant thoughts about quitting. Having many such thoughts is burdensome and can contribute to feelings of low self-efficacy.^12^ Feelings of guilt and shame seem to be common in people who continue to smoke after they are diagnosed with COPD; such feelings have also been reported in other studies.^13–15^ If not a chance finding, the positive relationship between pressure-filled mental states and reduced intensity of smoking in people not ready to try to quit might indicate that self-criticism and constant thoughts about quitting mark the beginning of a process of increasing awareness insufficient to lead to a quit attempt but sufficient to induce a reduction in the intensity of smoking behaviour. We also found it surprising that there were some indications that using destructive coping strategies to deal with pressure-filled mental states was associated with positive rather than negative cessation outcomes. Although the associations were not statistically significant, these tendencies warrant attention, and the relationships should be re-examined in larger studies. In fact, one should consider the possibility that answering the questionnaire *per se* increased the respondents’ awareness of these strategies and possibly of other factors that hinder successful quit attempts.

Indeed, the above-mentioned finding of similarity in smoking cessation trajectories between patients who reported being ready and those who reported not being ready to quit smoking at baseline suggests that the TTQ may be a better instrument for predicting cessation outcomes than a question about quitting intentions.

In patients who are not ready to try to quit, ambivalent thoughts seemed to be negatively correlated, although not significantly, with all the three outcomes. Ambivalence, usually defined as the simultaneous co-occurrence of a strong desire to smoke and a strong wish to quit smoking,^16^ is present in most smokers who want to quit. In this study, ambivalent thoughts (i.e., the desire to try new methods for quitting but a simultaneous worry about withdrawal symptoms, and the lack of desire to be informed about the progression of the disease) were reported in particular by patients who are not ready to quit. If confirmed in further studies, this finding provides important information for healthcare providers, for instance when taking a decision to use motivation-enhancing techniques in the counselling. In fact, the focus of motivational interviewing is exploring and resolving ambivalence.^17^

### Strengths and limitations of this study

The study was conducted in a naturalistic clinical setting, and very few patients declined to take part. The patients from all socio-demographic groups were included, the results can easily be generalised to the underlying regional population. The TTQ seemed to be easy to administer in the context of the nurse–patient interaction. An evident weakness of this study was the small sample size, which prevented us from drawing firm conclusions about the predictive ability of TTQ and especially of its subscales. A second limitation was that smoking habits were self-reported. A more valid measure of smoking reduction would have been obtained by measuring the CO levels in expired air.

These weaknesses notwithstanding, this study provided relevant information in two ways. First, it confirmed the importance of assessing, in the clinical setting, mental states connected with the process of quitting smoking. Second, it highlighted the importance of individualised goals in smoking-cessation counselling. For some people, reducing the number of cigarettes can be an achievable change and a step forward in the process of quitting.^18,19^

### Implications for future research, policy and practice

We suggest that the questionnaire should be further tested in larger samples and different cohorts as example patients with other chronic diseases before it can be generalised to a wider population.

### Conclusions

The TTQ questionnaire can identify specific mental processes related to successful cessation of smoking in COPD patients. Nurses and other healthcare providers can, therefore, use the brief TTQ questionnaire to support rational choices when counselling COPD patients who smoke.

## Materials and methods

### Design

The study was a longitudinal observational study of smokers with COPD recruited during planned and unplanned visits to primary healthcare centres in the Stockholm region between 2011 and 2013.

### Study population

**Figure 1:**
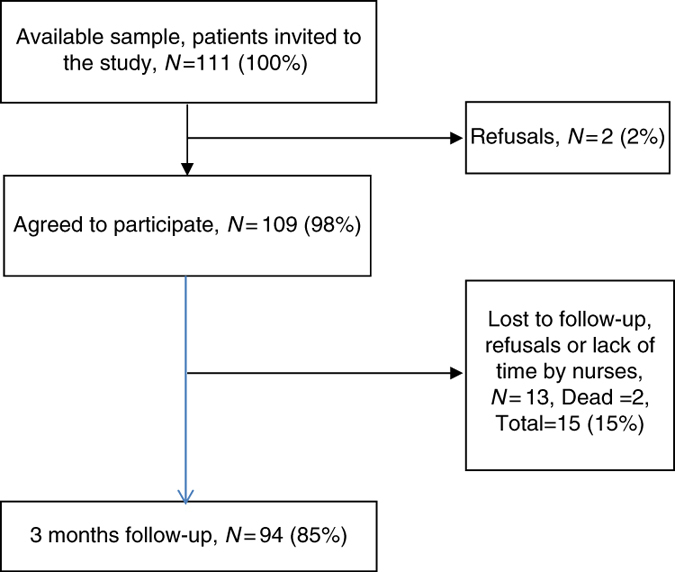
Patient inclusion, loss of patients and follow-up.

The study cohort consisted of 109 patients with COPD who were followed up for 3 months. The patients were recruited by 34 nurses at 31 primary healthcare centres; a diagnosis of COPD (using average forced expiratory volume during 1 s in %), being a current smoker and being able to speak Swedish were the eligibility criteria. The nurses provided the eligible patients with written information about the study and collected verbal consent to be enrolled. The baseline participation rate was 98% and the retention rate at the 3-month follow-up was 85% (Figure 1).

### Description of the questionnaire TTQ

There are two versions of the TTQ: an extended, original version and a revised, reduced version, which was created by removing five items from the original questionnaire. The longer, original version of the TTQ includes 19 items that measure aspects of the process of quitting smoking (mental states and strategies), likely to negatively impact the success of quit attempts in patients with COPD (Table 5).

As reported by Lundh *et al.* ^9^ we conducted an exploratory factor analysis with 14 items and found that three factors accounted for more than 90% of the variance and had a Cronbach’s alpha of 0.70%.^9^ We therefore removed five items from the original TTQ to create a shortened, 14-item questionnaire that has three sections (Table 3):

The responses to all the items on the TTQ are provided using a four-point Likert scale that ranges from 1 (do not agree at all) to 4 (agree completely). The item responses are summed to a total score, which ranges from 19 to 76 on the longer, original version of TTQ, and from 14 to 56 on the revised, brief version of TTQ.

The analyses are presented here both for the extended and for the reduced TTQ.

### Outcomes related to smoking cessation

The outcome variables were all self-reported and included the following: making at least a quit attempt that lasted at least 24 h, reducing the amount of smoking by 50% between baseline and the 3-month follow-up; and achieving complete abstinence during the 7 days before the follow-up assessment.

### Data collection

The 19-item questionnaire was administered at baseline by the nurses during a face-to-face interview with each patient. In addition, the patients completed a questionnaire on (i) demographic factors, family situation (living alone or with partner), time to diagnosis, severity of the disease and smoking history; (ii) cigarette dependence (Cigarette Dependence Scale);^20^ (iii) problematic alcohol use (Audit C);^21^ and (iv) anxiety and depression (Hospital Anxiety and Depression Scale).^22^ They also received a smoking cessation pamphlet and either brief advice on how to quit smoking or several sessions of more intensive smoking cessation counselling. The advice and counselling were not standardised, but followed nurses’ appraisals of the patients’ needs. A study record was kept for each patient. The record included type of smoking cessation support, time to complete the TTQ and the type of visit during which the TTQ was completed (unplanned visit, planned visit or visit to measure the lung function).

During the time between baseline and follow-up, the nurses registered all smoking cessation support for each patient on a special form. At the follow-up visit 3 months after baseline, the 19-item TTQ was administered again. In addition, nurses asked patients whether they had made any quit attempts lasting at least 24 h during the follow-up period, number of cigarettes usually smoked per day or per week if they had not quit and whether the patient had smoked at all in the 7 days before follow-up. In the analyses, reduction in smoking intensity was calculated by comparing the reported number of cigarettes smoked per day at follow-up with the reported number of cigarettes smoked per day at baseline.

### Statistical analysis

Continuous variables are presented as means±s.d. The Student's *t*-test and chi-square test were used to evaluate the baseline differences in demographic variables and the smoking history for continuous and categorical variables, respectively.

Unconditional logistic regression was used to measure ORs of making at least a quit attempt, reducing the intensity of smoking by 50%, or achieving smoking abstinence on the basis of total TTQ score. Adjustment for confounding variables was made by identifying potential confounders by *a priori* knowledge and testing the actual amount of confounding in multivariate models that included predictor and outcome variables. In the primary analysis with all the participants, the total score of the 19-item and 14-item TTQ were used. In the secondary analysis, separate analyses were run for two subgroups: patients ready to try to quit smoking and patients not ready to try to quit smoking. The patients were categorised into two groups through their answer to the question ‘What is your current decision about smoking?’ with the following response alternatives: (1) quit immediately, (2) try to quit as soon as possible, (3) put off trying to quit smoking or (4) continue to smoke’. These responses were then dichotomised into 1 to 2 and 3 and 4. Response alternatives 1 or 2 were categorised as ‘ready to quit’, whereas 3 or 4 indicated a patient ‘not ready to quit’.

IBM (Armon, NY, USA) SPSS statistics for Windows version 22.0 was used to conduct the data analysis. The level of statistical significance was set at <0.05.

### Ethical approval

The regional Ethical Review Board at Karolinska Institutet in Stockholm approved the study (2008/1929–31/5).

## Figures and Tables

**Table 1:** Demographic and behavioural characteristics of the participant patients

	*All participants, (*n*=109)*		*Participants ready to make a quit attempt,* n*=68 (62%)*		*Participants not ready to make a quit attempt,* n*=41 (38%)*		
	n	*(col %)*	n	*(col %)*	n	*(col %)*	*P value* a
*Gender*							
Men	34	(31)	21	(31)	13	(32)	
Women	75	(69)	47	(69)	28	(68)	0.93

*Education*							
Elementary school	49	(45)	32	(47)	17	(41)	
Upper secondary school	47	(43)	27	(39)	20	(49)	
University	12	(12)	9	(13)	4	(10)	0.63

*Family situation*							
Living alone	49	(45)	30	(44)	19	(46)	
Married/living with some one	59	(54)	37	(54)	22	(54)	0.87

*Time since diagnosis of COPD*							
⩽3 years	86	(79)	53	(78)	33	(80)	
>3 years	23	(21)	15	(22)	8	(20)	0.75
Other smokers in the social environment	65	(60)	26	(38)	23	(56)	0.60

*HADS depression (score range 0–21)*^b^							
Score ⩽7	84	(87)	54	(87)	30	(86)	
Score ⩾8	13	(13)	8	(13)	5	(14)	0.85

HADS anxiety (score range 0–21)^b^							
score ⩽7	58	(58)	37	(58)	21	(58)	
score ⩾8	42	(42)	27	(42)	15	(42)	0.96

Abbreviations: COPD, chronic obstructive pulmonary disease; HADS, Hospital Anxiety and Depression Scale.

a Comparison between participants ready to make a quit attempt and participants not ready to make a quit attempt.

b Missing values. Chi-square test. *P* value <0.05.

**Table 2:** Characteristics of the participants by age, severity of COPD, smoking habits, risky alcohol use and scores in TTQ

	*All participants,* n*=109*		*Participants ready to make a quit attempt,* n*=68*			*Participants not ready to make a quit attempt,* n*=41*			
	*Mean*	*(s.d.)*	*Mean*	*(s.d.)*	*Range*	*Mean*	*(s.d.)*	*Range*	*P value* a
Age	65	(7.7)	64	(7)	41–77	66	(8.8)	50–91	0.32
Average forced expiratory volume in 1 s in %^b^	60	(16.9)	58.2	(18.7)	18–105	64.1	(13.2)	37–96	0.45
Duration of smoking years at baseline	45	(8.3)	45.8	(6.8)	24–60	43.5	(10.2)	24–70	0.31
Daily smokers, cigarettes/day	13.9	(8)	13.2	(7.7)	0–30	15.2	(8.5)	1–40	0.21
Weekly smokers, cigarettes/week	18.3	(17.7)	19.7	(19)	1–40	10.0	(10)	10–10	0.22
Number of quit attempts	3	(4.2)	9.1	(17.7)	0–80	3.4	(5.1)	0–12	0.61
Cigarette dependence scale (score range 12–60)^b^	40.9	(7.9)	41.7	(7.4)	19–52	39.5	(8.6)	16–55	0.37
Risky alcohol use (score range 3–15)^b^,	6	(2.3)	6.3	(2.4)	3–13	5.5	(2.1)	3–10	0.49
TTQ 19 items (score range 19–76)	38	(5.3)	37.6	(5)	25–48	38.7	(5.7)	27–55	0.30
TTQ 14 items (score range14–56),	27.7	(4.9)	27.2	(4.9)	18–42	28.6	(4.7)	19–38	0.22
Developing pressure-filled mental states (score range 5–25)	11.3	(4.1)	10.5	(3.8)	5–20	12.5	(4.2)	5–20	0.16
Use of destructive pressure relief (score range 4–20)	5.5	(2.2)	5.7	(3.3)	4–14	5.1	(2)	4–14	0.53
Ambivalent thoughts (score range 5–25)	11	(2.3)	10.9	(2.4)	6–18	11.0	(2.2)	5–17	0.72

Abbreviations: CDS, cigarette dependence scale; TTQ, Trying To Quit smoking questionnaire.

a Comparison between participants ready to make a quit attempt and participants not ready to make a quit attempt is done by Student’s *t*-test.

b Missing values.

**Table 3:** The Trying To Quit smoking questionnaire (TTQ)—14 items

*Development of pressure-filled mental states (five items)*	*Ambivalent thoughts about quitting (five items)*	*Use of destructive pressure-relief strategies (four items)*
I feel criticised for not being able to quit smoking. I criticise myself for not being able to quit. I constantly think about quitting. I perceive it as failure that I am not able to quit smoking. I do not want show that I smoke.	I do not get support and encouragement when I try to quit smoking. I am worried about the way my body will react if I quit smoking. I am keen to try new methods as aids to smoking cessation. I do not want information about the progression of COPD.	It is unnecessary to quit because my health will not improve. It is unnecessary to quit because I am too old. It is unnecessary to quit because decreasing the number of cigarettes I smoke is sufficient. I do not feel that to quitting smoking is meaningful.

**Table 4:** Odds ratios and 95% confidence intervals of the relationship between smoking-related outcomes and increasing scores on the Trying To Quit Smoking questionnaire

	*All participants (*n*=109)*			*Ready to make a quit attempt (*n*=68)*			*Not ready to make a quit attempt (*n*=41)*		
	*Quit attempt,* a n*=36*	*Reducing amount of smoking by 50%,* b n*=31*	*Achieving complete abstinence,* b n*=17*	*Quit attempt,* a n*=21 (31%)*	*Reducing amount of smoking by 50%,* b n*=18 (26%)*	*Achieving complete abstinence,* c n*=10 (15%)*	*Quit attempt,* a n*=15 (37%)*	*Reducing amount of smoking by 50%,* b n*=13 (32%)*	*Achieving complete abstinence,* b n*=7 (17%)*
	*OR (95% CI)*	*OR (95% CI)*	*OR (95% CI)*	*OR (95% CI)*	*OR (95% CI)*	*OR (95% CI)*	*OR (95% CI)*	*OR (95% CI)*	*OR (95% CI)*
Total TTQ score all items continuous (19 items)	0.90* (0.83–0.98)	0.97 (0.89–1.05)	0.92 (0.82–1.01)	0.92 (0.83–1.02)	0.98 (0.88–1.09)	0.96 (0.84–1.09)	0.83* (0.70–0.98)	0.93 (0.82–1.06)	0.80 (0.64–1.00)
Total adjusted TTQ score (19 items)^d^	0.89* (0.80–0.98)	0.90 (0.87–1.02)	0.91 (0.81–1.02)	0.91 (0.80–1.04)	0.96 (0.85–1.09)	0.93 (0.80–1.09)	0.78 (0.62–1.00)	0.88 (0.74–1.05)	0.72* (0.53–0.99)
Revised TTQ total score (14 items)^e^	0.94 (0.86–1.03)	0.97 (0.88–1.06)	0.98 (0.88–1.09)	0.94 (0.84–1.05)	1.04 (0.93–1.16)	0.96 (0.84–1.11)	1.02 (0.88–1.17)	1.18 (0.99–1.39)	1.09 (0.90–1.30)
Pressure-filled mental states (5 items)^e^	0.90 (0.81–1.01)	1.11 (1.00–1.25)	0.98 (0.87–1.12)	0.78* (0.66–0.94)	1.03 (0.89–1.19)	0.86 (0.70–1.06)	1.01 (0.86–1.20	1.32* (1.05–1.66)	1.11 (0.91–1.36)
Destructive pressure-relief strategies (4 items)^e^	1.18 (0.98–1.43)	1.10 (0.91–1.33)	1.27 (1.04–1.56)	1.17 (0.94–1.46)	1.09 (0.86–1.37)	1.28 (0.99–1.65)	1.57 (0.78–3.16)	1.23 (0.75–2.00)	1.44 (0.88–2.34)
Ambivalent thoughts (5 items)^e^	0.99 (0.82–1.18)	0.94 (0.78–1.13)	0.81 (0.63–1.04)	1.06 (0.85–1.33)	0.99 (0.78–1.24)	0.89 (0.65–1.20)	0.82 (0.58–1.15)	0.78 (0.54–1.12)	0.66 (0.41–1.06)

Abbreviations: CI, confidence interval; OR, odds ratio; TTQ, Trying To Quit smoking questionnaire.

a Quit attempt= making at least one quit attempt (yes/no).

b Reducing amount of smoking by 50%=50% reduction in the number of cigarettes smoked per day.

c Achieving complete abstinence=achieving complete abstinence for the 7 days before follow-up.

d Adjusted for gender, education, total cigarette dependence scale score and smoking cessation counselling.

e Adjusted for smoking cessation counselling. *P value <0.05.

**Table 5:** The Trying To Quit smoking questionnaire (TTQ)—19 items

*Items*	
1. I plan to quit smoking. 2. I try to quit smoking. 3. I feel criticised for not being able to quit smoking. 4. I criticise myself for not being able to quit. 5. I do not get support and encouragement when I try to quit smoking. 6. I feel worried about consequences if I do not quit smoking. 7. I am worried about physical reactions if I quit smoking. 8. I feel that I must quit smoking. 9. I constantly think about quitting smoking. 10. I perceive it as a failure not being able to quit smoking.	11. I am keen to try new methods as aids to smoking cessation. 12. I do not want information about the progression of COPD. 13. I do not want to show that I smoke. 14. I have difficulties to stop smoking because my husband/wife/friend smokes. 15. It is unnecessary to quit because my health will not improve. 16. It is unnecessary to quit because I am too old. 17. 1t is unnecessary to quit because decreasing the number of cigarettes is sufficient. 18. I hope to be able to quit smoking some day. 19. I do not feel that to quit smoking is meaningful.
